# Exploring culturally-preferred communication approaches for increased uptake of voluntary medical male circumcision (VMMC) services in rural Malawi

**DOI:** 10.1186/s12889-023-15363-x

**Published:** 2023-03-29

**Authors:** Kent Yelemia G. Mphepo, Adamson Sinjani Muula, Joel Suzi, Felix Phuka, Joseph Mfutso-Bengo

**Affiliations:** Department of Public Health, Kamuzu University of Health Sciences (KUHeS), Blantyre, Malawi

**Keywords:** Theory, Source, Message, Audience, Channel, Effects, Feedback

## Abstract

**Background:**

In 2007 WHO and UNAIDS recommended communication interventions as a key strategy for creating demand for Voluntary Medical Male Circumcision (VMMC) in Southern Africa. In Malawi, VMMC communication interventions, implemented by health communication agencies, have effectively raised awareness of services. However, high awareness of VMMC has not resulted in increased uptake. Consequently, Malawi has achieved the lowest number of circumcisions in Southern Africa.

**Methods:**

These researchers carried out a study among the traditionally circumcising Yaos of Mangochi in Southern Region and the non-circumcising Chewas in Central Region. Data were collected using FGDs, KIIs, IDIs, Life Histories and Participatory Rural Appraisal methods. Data were analyzed thematically.

**Results:**

This study demonstrates two lessons. First, Laswell’s Theory, which has traditionally been used in politics, is relevant to the health sector where the message delivery continuum also needs to be clear on source, message, audience, channel and intended effects. Secondly, according to informants, allowing communities to give *feedback* to the VMMC messages delivered by health promoters is fundamental. Therefore, failure by Laswell Theory to emphasize on feedback compromises its efficacy. It weakens its ability to foster a *common vision* between the *source* and the *audience* which is prerequisite for behavioral change.

**Conclusion:**

The study concluded that *community engagement* and *interpersonal communication* which provide room for real-time *feedback* in any communicative event are the most preferred communication interventions for VMMC services among Yaos and Chewas.

## Introduction

In 2010, approximately 54, 000 new HIV infections were registered in Malawi. In response to this alarming figure, Malawi committed to reducing new infections by 75% by 2020 [1, 2]. By that year, Malawi had registered 19,000 new infections [2]. In light of Malawi’s prevailing HIV burden, voluntary medical male circumcision (VMMC) became a key HIV prevention strategy as per joint World Health Organization (WHO) and The Joint United Nations Programme on HIV/AIDS (UNAIDS) recommendation of 2007 [2]. Supported by evidence from three randomized studies conducted in South Africa, Uganda and Kenya which revealed that circumcision was effective in reducing HIV transmission among heterosexual men by 60–70%, in 2007, both UNAIDS and WHO recommended VMMC as a new HIV prevention measure [1, 2]. The recommendation specifically targeted 14 countries with high HIV burden in the sub-Saharan Africa region, including Malawi [1–3].

Globally, by 2019 nearly 26.8 million cumulative male circumcisions for HIV prevention were performed between 2008 and 2019 in the 15 priority countries of East and Southern Africa [4]. This number of circumcisions had averted about 340,000 new cases of HIV by 2019[4]. As a result of the registered success, it was projected that by 2030 some 1.8 million new infections would be prevented [5]. In the same period, Malawi performed cumulatively 887,205 medical circumcisions representing approximately 31% of the target for 2020 [5].

As in the case of Uganda, Malawi’s VMMC implementation faced some hesitancy due to among others, lack of local evidence on benefits of the intervention [5, 6]. Malawi developed its first national policy on VMMC in 2012, which was incorporated into a mix of HIV prevention strategies in the country [2, 7]. However, performance towards the set targets has generally been slacking [2]. With VMMC prevalence currently at 31% of men aged between 15 and 49, Malawi trailed the 60% target by almost 50% [2].

Communication has been integral to VMMC implementation and specifically in VMMC demand creation in Malawi [2, 7]. The first VMMC communication strategy in Malawi was implemented from 2012 to 2016 [2, 8–10]. The 2010 Situation Analysis Report recommended a VMMC communication strategy to create demand for services [2, 6, 7, 11, 12]. At regional level, findings of a study in Kenya adds weight behind communication as a tool for increasing VMMC uptake [13]. Accordingly, a communication strategy was embedded into the implementation package for VMMC for purposes of demand creation [2, 7, 10].

Malawi Government acknowledges that VMMC demand creation through communication interventions has proved challenging [2]. Though there is a high awareness of VMMC’s health benefits among the target audience, there is low uptake among both circumcising and non-circumcising communities of Malawi [2, 7, 14, 15]. Several studies have shown that most tribes and religious groups regard circumcision as ‘amoral’ and ‘intrusive’ to their social-cultural values, beliefs, and traditions; thus, also viewed as ‘threatening’ their identity of tribes and religious groups [16–18]. In addition, fear of pain, the long recovery period, perceived fertility loss, and medical complications are some other factors fueling the resistance to medical circumcision among adults [1, 6, 11, 15, 19]. The impact of these factors on uptake among adult men in Malawi suggests the need for efforts to increase uptake [6].

Communication is integral to countering these factors by boosting knowledge, understanding and positive attitudes toward the service and in turn stimulating demand and uptake among Malawian men, boys, parents/guardians and partners [2]. Hence, the Malawi government developed the first VMMC Communication Strategy in 2012 to guide design and implementation of strategic communications within the framework of the national VMMC policy towards achieving the 80% uptake of VMMC among eligible males aged 10–49 by 2025 [2, 20].

## The status of HIV/AIDS and VMMC in the Sub-Sahara Africa region

By 2007, the Sub-Sahara African Region had the highest HIV prevalence globally and had very low male circumcision prevalence allegedly due to the widespread presence of Christianity which did not encourage male circumcision and the historical legacy of British colonialism [21]. Following three randomized clinical trials in Uganda, Kenya and South Africa in 2005, in 2007 WHO and UNAIDS recommended to 14 East and Southern Africa countries, to take VMMC as an extra arsenal in the fight against HIV transmission [22]. These countries included Botswana, Ethiopia, Kenya, Lesotho, Malawi, Mozambique, Namibia, Rwanda, South Africa, Swaziland, Uganda, United Republic of Tanzania, Zambia and Zimbabwe [23]. By 2017 nearly 15,269,720 million boys and men had been circumcised in a decade with support from PEPFAR alone and figures have continued to rise [24].

## The status of HIV/AIDS and VMMC in Malawi

In 2013, UNAIDS estimated that the world had 35 Million people living with HIV; in the same year, 2.1 million more people got infected and 1.5 people died of AIDS-related illnesses [25]. In Malawi, HIV prevalence among persons aged between 15 and 49 has been reducing from 16.4% in 1999 to 8.5 in 2020 [26]. Overall, new infections among the 15–49 brackets were reducing. While in 2011 the country registered 55,000 new infections, it only registered 19, 000 in 2020 [26]. This, however, remains an unacceptably very high number compared to the population size of approximately 19 million people and calls for concerted effort in a drive to cut the number of new infections to 0 by 2030 [26]. Although national average adult HIV prevalence hovered around 8.5% the country had witnessed about 10, 000 HIV-related deaths at the end of 2020 [26].

## Challenges facing communication on VMMC services in Malawi

In Malawi, Male Circumcision is most common in Southern Region with prevalence rate at 47% among men aged 15–49 seconded by Central Region at 15% and lastly at 6% in the Northern Region of the country [27]. By 2018, overall VMMC performance in the country hovered around 30% of the total target population of males in the age bracket of 15 to 49 [20]. According to available data, low performance of VMMC in Malawi could be attributed to social-cultural factors and perceived adverse effects following the procedure at social-ecological level [2, 20]. Specifically, VMMC was perceived by both traditionally circumcising and non-circumcising ethnic groups as a threat to cultural identity, religious beliefs and values [2, 20]. At personal level, males avoided VMMC for fear of being tested for HIV at the health facility; concerns of meeting travel costs to the health facility; concerns over losing income during the recuperation period; fear of infidelity on the other partner among married couples either during the recuperation period (on the part of males) and after recuperation (on the part of females); unwillingness to abstain from sexual activity six weeks after undergoing the VMMC procedure and perception that VMMC diminishes sexual pleasure [2, 20].

Related to VMMC communication, Mhagama et al had concluded that although VMMC was purportedly voluntary, most up-takers did not do so voluntarily [12]. According to the study that was conducted in Lilongwe, men were influenced to uptake VMMC mostly by peer pressure and the need for conformity; partner/girlfriend demand and considerations; and advice from health personnel [12]. That men opt for VMMC under duress impedes on VMMC uptake [12]. It is therefore the task of communication to bring to the fore the salience of voluntariness in VMMC in order to increase uptake among men.

## Conceptual framework

The word “communication” finds its roots in the Latin word, “communis” which translates “common”. The key purpose of communication interventions is to create a common understanding between the communicator and the audience [28]. Linking “communication” to behavior change, Carl Hovland defines communication as:

*“…the process by which an individual (the communicator) transmits stimuli (usually verbal symbols) to modify the behavior of the other individuals (communicates)”* [28].

**Fig. 1:**
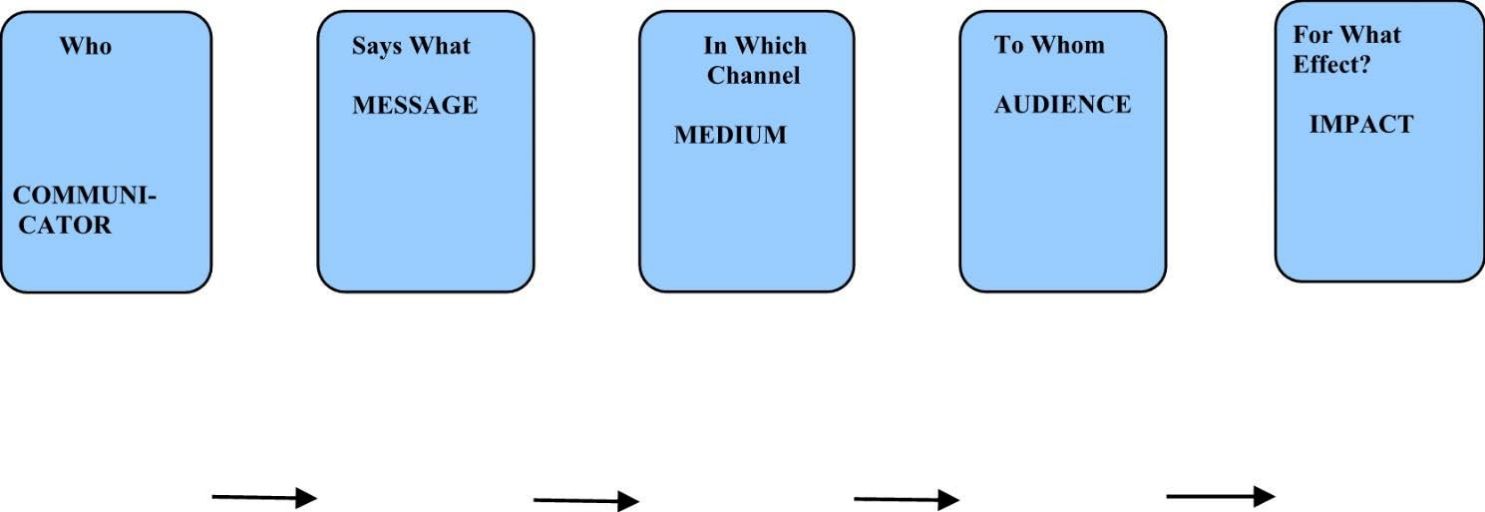
A typical conception of Laswell’s Construct as a graphic model of communication [29]

In order to effectively identify the preferred communication approaches for the promotion of VMMC services among the Yao of Mangochi and the Chewa of Dowa in Southern and Central regions respectively, these researchers used Laswell’s Transmission Theory which is anchored by the dictum “Who says What, in Which Channel, to Whom and for What Effect?” (as indicated in Fig. 1: A typical conception of Laswell’s construct as a graphic model of communication**)** [29]. This theory was named after Harold Dwight Laswell an American political scientist and a communication theorist who in 1948, while he was a professor at Yale Law School, wrote in his article entitled: *“The Structure and Function of Communication in Society”*, [30]:

*…the most convenient way to describe an act of communication is to answer the following questions: a. Who b. Says What c. In Which Channel d. To Whom e. With What Effect? We call that the “5Ws” model* [29].

This framework effectively divides up the field of communication into five key areas of study and research. These researchers framed questions from these five fields to unearth the preferred communication approaches to be used by health communicators in both circumcising and non-circumcising communities. Informants answered these five most important questions: (a) What is your preferred *source* of information for VMMC information? (b) What is your preferred *message* for VMMC communication interventions? (c) What is your preferred *audience* for VMMC messaging? (d) What is your preferred *channel* for VMMC communication interventions? (e) What are your expected *effects* (or impacts) of VMMC communication interventions? When data were collected and analyzed these researchers noted, just like other scholars have, that *feedback* is the key missing element in Laswell’s 5Ws Model since communication is a two-way and not a one-way road [30]. They noted through this study too that current communication interventions meant to promote VMMC services in Malawi did not pay adequate attention to these five elements. These researchers also discovered that, in Malawi, the most popular and preferred communication approaches were *community engagement* and *interpersonal communication* since they allowed the audience to give real-time *feedback* to communicators on VMMC messages.

## Method

This study was informed by the works and commentaries on the Social-Ecological Model, Laswell’s Transmission Theory, and numerous communication and behaviour change theories. Secondly, investigators collected data among the predominantly circumcising Yao of Mangochi and the predominantly non-circumcising Chewa of Dowa. Researchers used FGDs, KIIs and IDIs to gain a comprehensive and deep understanding of the values, beliefs and traditions that underlie the resistance to VMMC. Data were thematically analyzed to decipher the people’s communication needs and preferred communication approaches by VMMC campaigners.

### Sample population

Total number of participants in this study was 276 (160 males and 116 females. We conducted 24 focus group discussions (FGDs); 13 in Mangochi involving 118 participants (66 males and 52 females), 11 in Dowa involving 97 participants (54 males and 43 females). This makes a total of 215 participants for both Mangochi and Dowa. There were 9 key informant interviews involving 14 participants 4 interviews of Mangochi (7 males 0 females) and 5 interviews in Dowa involving 7 participants (4 males and 3 females). There were 16 in-depth interviews, involving 19 participants (14 males and 5 females). In Dowa there were 7 in-depth interviews involving 10 people (6 males and 4 females). In Mangochi there were 9 in-depth interviews involving 10 participants (9 males and 1 female). There were 5 life histories involving 5 participants (2 in Dowa and 3 in Mangochi). In Dowa 1 was male and 2 were females. In Mangochi there was 1 male and 1 female. There were two PRAs involving 23 people. In Dowa there were 11 participants (6 males and 5 females). In Mangochi there were 12 participants (6 males and 6 females) The interview guides during this study were based on the five key questions as indicated in Fig. 2 below. By collecting data from 160 males and 116 females including youths and minors, we explored the social and cultural values that influence people’s decisions to seek health care. Specifically, the study explored the reasons why uptake of VMMC services was low. We also explored the preferred communication approaches that VMMC campaigners could use in order to motivate men and boys to access services. Figure 3 below shows the details of data collection channels disintegrated according to districts, research tools and participant categories.

**Figure 2:** Key Domains, Target Groups, and Illustrative Questions for Interviews and Focus Groups

Domains	Target Groups	Illustrative Key Questions
1. Preferred **‘Source’**of VMMC information	Focus Groups, Key informants and in-depth interviews, Life Histories and Participatory Rural Appraisal methods	In this area, what is the preferred source of information for VMMC information?
2. Preferred **‘Message’** for VMMC Information	Focus Groups, Key informants and in-depth interviews, Life Histories and Participatory Rural Appraisal methods	In this area, what is the preferred message for VMMC communication interventions?
3. Preferred **‘Channel’** of VMMC information	Focus Groups, Key informants and in-depth interviews, Life Histories and Participatory Rural Appraisal methods	In this area, what is the preferred channel for VMMC communication interventions?
4. Preferred **‘Audience’** for VMMC information	Focus Groups, Key informants and in-depth interviews, Life Histories and Participatory Rural Appraisal methods	In this area what is the preferred audience for VMMC messaging?
5. Preferred **‘Effect’** of VMMC information	Focus Groups, Key informants and in-depth interviews, Life Histories and Participatory Rural Appraisal methods	In this area, what are the expected effects (or impacts) of VMMC communication interventions?

**Figure 3:** Table of data collection categories

Tool	Community	District	Age Range	Sex		Participants
			**Focus Group Discussions (FGD)**			
				**Male**	**Female**	
FGD - Adult men	Kapinjiri	Mangochi	22–37	6	0	6
FGD – Unmarried youth	Chowe	Mangochi	17–19	6	6	12
FGD – Adult men	Chowe	Mangochi	38–55	11	0	11
FGD – Adult Women	Mkundi	Mangochi	30–45	0	7	7
FGD – Adult women	Chowe	Mangochi	18–47	0	12	12
FGD – Traditional	Chowe	Mangochi		3	3	6
FGD – Gate Keepers	Chowe	Mangochi	26–70	6	5	11
FGD – Married Youth	Chowe	Mangochi		6	6	12
FGD – New Initiates	Chowe	Mangochi	8–13	11	0	11
FGD – Religious Leaders	Jalasi	Mangochi		10	0	10
FGD - Commercial Sex Workers	Mangochi	Mangochi		0	6	6
FGD – Service Providers	Jalasi	Mangocgi	8	4	4	8
FGD – Service Providers	Chowe	Mangochi	26–29	3	3	6
						0
FGD – Adult women	Mwancheka	Dowa	27–78	0	12	12
FGD – Gate Keepers	Mwancheka	Dowa	20–67	9	2	11
FGD - Married Youth	Mwancheka	Dowa		6	4	10
FGD – New initieates	Mwancheka	Dowa	12–16	4	3	7
FGD – Religious Leaders	Mwancheka	Dowa		10	1	11
FGD – Service Providers	Khuwi/Kadewa	Dowa	28–48	2	4	6
FGD – Service Providers	Mponela	Dowa	28–56	4	3	7
FGD – Sex workers	Mponela	Dowa	29–36	0	6	6
FGD – Traditional Leaders	Mwancheka	Dowa	25 52	6	1	7
FGD – Unmarried Youth	Mwancheka	Dowa	14–29	6	7	13
FGD – VMMC Clients	Mwancheka	Dowa		7	0	7
			**Total**	**120**	**95**	**215**
			**Dowa**	**54**	**43**	**97**
			**Mangochi**	**66**	**52**	**118**
			**In-Depth Interviews (IDI)**			
IDI - Ngaliba	Chowe	Mangochi		**1**	**0**	**1**
IDI - Sheikh	Lilongwe			**1**	**0**	**1**
IDI - Sheikh	Chowe	Mangochi		**1**	**0**	**1**
IDI - Pastor	Chowe	Mangochi		**1**	**0**	**1**
IDI - Nakanga (Female)	Chowe	Mangochi		**0**	**1**	**1**
IDI - Nakanga (male)	Chowe	Mangochi		**1**	**0**	**1**
IDI - Alhaji	Jalasi	Mangochi		**1**	**0**	**1**
IDI - Traditional Leader	Chowe	Mangochi		**1**	**0**	**1**
IDI - Catechist	Jalasi	Mangochi		**1**	**0**	**1**
IDI - Traditional Leader	Dowa	Dowa		1	0	1
IDI - Pastor CCAP	Mponela	Dowa		1	0	1
IDI - Gulewamkulu	Dowa	Dowa		1	0	1
IDI - Counsellors - Catholic	Mwancheka	Dowa		1	1	2
IDI - Counsellors CCAP	Mwancheka	Dowa		1	1	2
IDI - Counsellor - Anglican	Mwancheka	Dowa		0	1	1
IDI – Community members	Mwancheka	Dowa		1	1	2
				**14**	**5**	**19**
			**Life Histories (LH)**			
LH – Nakanga	Chowe	Mangochi			1	1
LH – Ngaliba	Chowe	Mangochi		1	0	1
LH - Church Counsellor	Mwancheka	Dowa		0	1	1
LH - Female Gulewankulu Leader	Mwancheka	Dowa		0	1	1
IDI - Wakunjira	Mwancheka	Dowa		1	0	1
			Total	**2**	**3**	**5**
			**Key Informant Interviews**			
KII – Culture Experts	Chowe	Mangochi		2	0	2
KII - Service Providers	Mangochi	Mangochi		2	0	2
KII - Service Provider	Chowe	Mangochi		2	0	2
KII - Traditional Leader	Chowe	Mangochi		1	0	1
KII - Expert	Mwancheka	Dowa		1	0	**1**
KII - Expert	Mwancheka	Dowa		1	0	**1**
KII - Expert	Mwancheka	Dowa		1	0	1
KII - Sex Workers	Mponela	Dowa		0	3	3
KII - Traditional Leader	Mwancheka	Dowa		1	0	1
			**Total**	**11**	**3**	**14**
		**Participatory Rural Appraisal (PRA)**				
PRA -	Chowe	Mangochi		7	5	12
PRA – Community	Mwancheka	Dowa	15–49	6	5	11
			**Total**	**13**	**10**	**23**
			**Total Participants**			**276**
			**Female Participants**			**116**
			**Male Participants**			**160**

Key: MHG = Mangochi; DA = Dowa; FGD = Focus group discussion; IDI = In-Depth Interview; KII = Key Informant Interview; LH = Life History; PRA = Participatory Rural Appraisal

## Findings

Findings of this study revolved around the five critical questions of transmission inquiry as suggested by Harold Laswell namely *‘who says what, to whom, through what channel, and with what effect?*’ Five questions framed the presentation of results namely: (a) What is the preferred source of information for VMMC information? (b) What is the preferred message for VMMC communication interventions? (c) What is the preferred channel for VMMC communication interventions? (d) What is the preferred audience for VMMC messaging? (e) What are the expected effects (or impacts) of VMMC communication interventions?

### Communication ‘noise’ behind the low attention to information on VMMC services in the two study sites

We first report on the cultural and religious factors that impeded VMMC communication in the two data collection sites. Overall, this study found that royalty to culture and religion prevented people from the two study sites from acting on messages. These messages were being disseminated by various VMMC implementing agencies, predominantly NGOs, with support from various development partners, under the leadership of the Ministry of Health. However, the traditionally circumcising Yao people avoided VMMC services on the fear that it undermined jando, a cultural initiation ritual where boys were circumcised to symbolize transition from childhood to adulthood. VMMC messages and the VMMC program as a whole, were perceived to be in conflict with their cultural identities and, therefore, a threat to the survival of their initiation practices which, for the circumcising Yaos, went beyond the mere cutting of the foreskin but also inculcated character in the initiates: *“Ours was the circumcision of the brain not of the penis as they are portraying it now… As Yaos we were circumcised for cleanliness, moral discipline and transition to manhood,”***[KII, Yao Culture Expert**^1^**].** Jando also had a political significance since boys were prepared for fiduciary duties towards their local chief and enabled them to practice a ritual that was sanctioned by Islamic Scriptures.*“…This time around you are telling the chiefs that circumcision should be done at the hospital. The chief cannot promote this because he is benefiting nothing ….,”* [**KII, Yao Cultural Expert & Sheikh].**

On the other hand, being a predominantly traditionally non-circumcising and Christian ethnic group, the Chewa people did not pay much attention to VMMC messages. Their understanding was twofold: first, male circumcision was not part of their culture. Secondly, male circumcision was not part of their religion but an attempt by government to popularize Islam in their area. Informants, therefore, equated getting circumcised with becoming a Yao or a Muslim. One man said: “*I am a Chewa man, why should I go for circumcision? Do you want me to become a Yao?”* Another one was more conscious of his faith: “*As Christians, we do not make [put] emphasis on circumcision,”***[IDI, Religious leader - Dowa].** These researchers posit, therefore, that cultural and religious perception, accounted for low uptake of VMMC services in the two data collection communities.

### Culturally-preferred communication approaches based on Laswell’s theory

Based on Harold Laswell’s fivefold question we now present results of the study as follows:

#### What is the preferred source of information for VMMC information?

Both cultures under study perceived the chief as a primary source of information and a mouth-piece of the community. Hence any information to be delivered to the community needed to pass through and vetted by him or her. Despite that among the Yao allegiance was determined by one’s economic standing, among the Chewa it was more of the cultural royalty. From that end, the study informants first noted that promoters of VMMC had overlooked this important community entry protocol thereby creating a communication barrier.

*“They must tell the chief, [and] the chief will announce to his people that the hospital personnel have something to say, so everyone will go to the meeting…The chief will open the floor and hand it over to the hospital staff to address the people.”***[FGD, Unmarried Youth – Dowa].**

From the foregoing, the chief was traditionally, the primary source of information. However, for lack of technical knowledge and skills on the subject of VMMC services study participants in both communities identified health workers as a **preferred source** of information. Study participants held that health workers were well trained hence knowledgeable, experienced and holding reliable information. *“…medical personnel should be assigned to spread the message of circumcision because they are well trained. If they were to convey the message it will be better understood,”***[IDI, Traditional Leader – Dowa].**

#### What is the preferred message for VMMC communication interventions?

The most **preferred message** that communities want to hear from those implementing VMMC interventions is benefits that VMMC offers to communities, households, and men and boys in particular. Regarding pre-adolescents, parents, clan leaders, community and religious leaders from Mangochi district indicated that they needed to be schooled on how VMMC provided long-term health benefits to males. For example, they needed to learn about such long-term benefits as the reduction of the risk of contracting HIV during traditional circumcision rituals during *ndagala*.^2^ Parents and guardians also wanted assurance that VMMC offered their wards protection against HIV infection during heterosexual relationships before and after they began to raise their future families. An unmarried youth in Dowa narrated as follows:

“*I think the most vital information is that people should know the benefits… of circumcision and to my understanding circumcision primarily protects you from cervical cancer so if they are told the women will encourage the men to go,”* [**FP1, Dowa Unmarried Youth].**

A female respondent in Dowa was of the view that men were not provided with sufficient details regarding VMMC procedure. From her perspective, VMMC messages needed to clearly explain what exactly made a circumcised person different from a non-circumcised man. She observed that VMMC messages had scanty details on what the circumcision procedure involved and what really happened to the man. She argued that men, who were the target of opportunity for VMMC interventions, were blank on details of the procedure:

*“…We just said they circumcise each other but we did not really know what they really cut. People just say the ‘foreskin’ of the penis others just say the ‘[head of the] penis’ so when you think about it, eeh! It’s scaring.”***[FGD, Gate Keepers - Dowa].**

The study established that the role of VMMC in the prevention of cervical cancer was more attractive than the role that it played in the reduction of HIV infection since everybody in the area knew at least one woman who had died of cervical cancer. They, therefore, wanted messages to focus on how VMMC would protect females from cervical cancer.

*“The protection from cervical cancer in women is most important in this area…we don’t have the desire for promiscuity and our women don’t have the desire of seeking sexual satisfaction in bed from circumcised men… Let us put HIV aside, currently the lives of people are at risk. You can manage HIV when infected, but there is no cure for cancer,”***[FGD, Traditional Leaders - Dowa].**

Female participants wanted VMMC messages to focus more on how their sons would be circumcised by trained medical personnel, which would result in less pain, loss of blood and reduce the risk of complications.

*“…in the past we had STIs like chindoko*^3^*mabomu*^4^*but they were treatable but these days there is AIDS which is very dangerous. There is [cervical] cancer and chisonono*^5^*too. VMMC is helpful since we hear that it protects our youth and their [future] families from these. These are messages that we need hear about,”***[FGD, Adult Women – Mangochi].**

VMMC messages also needed to fight against stigma and must assure both Christians and Muslims that VMMC did not contradict the Bible and the Qur’an respectively. One Sheikh, for example, was pleased with VMMC, as a religious authority:

*“…when I look at the current situation as an Islamic leader, I am very pleased because what is happening now [in clinics] is real jando as prescribed by the Islamic faith.… VMMC fully agrees with Islam.”***[KII, Religious Leaders - Mangochi]**

#### What is the preferred channel for VMMC communication interventions?

Regarding the **channel of communication** for VMMC intervention, many informants reported that the media, particularly radio was prominently used in both study communities.

*“…to avoid the huge cost [that would come with village meetings] then I can only agree with the media approaches that are being used currently… just think about how MBC Radio One is boasting that 87% of the Malawi population are able to hear them… Now if 87% are listening to MBC [alone] how about those listening to Zodiak?”***[IDI, Religious Leader – Mangochi].**

However, not many informants were excited about the use of such open channels to promote male circumcision both in Dowa and Mangochi district. In the two districts the use of open media channels was frowned upon for infringing on the secrecy that surrounds rites of passage rituals. First, male circumcision among the Yao was not a subject for public discussion. Second, although both the Quran and the Bible legitimized it, the praxis in the Muslim Yao community was to veil it in secrecy since those not circumcised and females, in particular, were not supposed to know what happened out in the bush:

*“In the past, circumcision was a total secret such that even the boy’s mother did not know why her child was going to ndagala*^6^. *The majority of the mothers celebrated jando blindly. And when we were there they strictly told us not to tell anybody what had happened to us. They told us that when we went back home we must bath with pants on lest the mother notices the difference in the appearance of the member….”***[KII, Religious Leaders - Mangochi].**

Another key concern with the use of radio, television and other forms of mass media was the sexual explicitness that such messages were associated with. This concern was particularly common among adult men and women who feared that such messages would have negative consequences since they promoted sexual immorality among the youth. This, they argued, was contrary to the purpose of male circumcision in the Yao culture:

*“The problem comes in because … [through the media] you are telling people that once you have been circumcised, you are free from HIV. These are wrong messages…You will [now] hear a man telling a woman that ‘I was circumcised you can come for sex I can’t contract diseases,’”***[KII, Yao Culture Expert - Mangochi].**

Informants’ most preferred communication channel both among the Yaos of Mangochi and the Chewas of Dowa district was face-to-face engagement meetings attended by health promotion experts on one hand and community leaders, cultural gatekeepers and community members on the other. Among these methods were: meetings where traditional leaders would bring their subjects to one place and engage them in a dialogue; cultural performances such as *gule wankulu;* religious gatherings such as regular weekly services of worship; interactive drama and door-to-door visits just to mention some. The key reasons for preference were twofold: first, unlike the mass media, they provided room to control the type of audience to hear the message or not. Secondly, they provided an opportunity for the audience to ask questions and seek clarifications as messages are delivered. Engagement meetings organized by traditional leaders were important since chiefs and their councils of advisors acted as a bridge between citizens and government and its stakeholders:

*“… gulewankulu characters can dance then the drama can come next to teach things like these or you can introduce the topic at first so that the people can have an idea so when the drama on circumcision comes it will entertain them,”***[FGD, Gatekeepers – Dowa].**

Although informants from all Protestant Christian churches including the Catholic leadership in Dowa sounded very negative about using the church to promote VMMC messages, in Mangochi the Catholic Church was already disseminating VMMC messages. I was also carrying out surgeries. One *gulupa*^7^ said: “*… [Children] are also assisted to get circumcised. When they get to that stage, they are kept at the parish, which is like a simba*^8^*….”*

In Chowe area, Mangochi, FGD participants proposed that VMMC messages should be disseminated through their mosques and churches. This is how one traditional leader put it: *“In this community, Churches and Mosques are more ideal. Religious gatherings are easy places to disseminate VMMC information,”***[FGD, Traditional Leaders, Mangochi].**

In Mangochi, a representative of an NGO added that they used males from within the community to deliver VMMC messages to fellow men. The health worker posited that men tended to listen better to health messages when delivered by their peers. “*We have community mobilizers who are recruited from within communities. They reach out to men in the communities,”***[KII Participant, NGO Worker – Mangochi].** Another NGO worker said: *“Our main approach with demand creation is interpersonal communication.”***[KII Participant, NGO Worker - Mangochi]**.

#### What is the preferred audience for VMMC messaging?

The study established that VMMC messages should target all community members including men, women and boys to avoid misunderstandings that may arise from misinterpretation of the messages particularly among married couples. This is what one IDI participant said:

*“These days I think that everyone [should be targeted] because, in a family, the man may hear the message alone but when he brings the message into his household the wife may misinterpret it… If the message was received by all - the man, the woman and the boy … people would easily embrace it,”***[IDI, Traditional Leader – Dowa].** This view was supported by another community leader who argued that the message must go to both males and females to avoid disagreements between married couples: *“The message has to go to both because the women are the ones who stay with the children. They can get them circumcised,”***[FGD, Gatekeepers – Dowa].**

The study also established that another key entry point into VMMC among the Yao men of Mangochi was the pre-adolescence age bracket of 5 to 10 years. They were minors such that they needed consent from their parents or guardians or those in authority of a village of institution. Two KII participants in Mangochi suggested that VMMC messaging must also target teachers and schools: *“…I think that teachers also are very important in their own right because they interact with the community [and learners] in particular. I have a view that teachers can help us clear a lot of misconceptions …,”***[KII, Service Providers – Mangochi].**

This view point agrees with the observation that one participant in an FGD with Service Providers made on the same matter in Dowa. He lamented that although schools had a lot of HIV/AIDS-related clubs authorities had failed to utilize them to popularized VMMC services among adolescents and youth.

*“At school we have HIV&AIDS youth clubs but because they were not oriented on [VMMC] services … it is hard for you [as a teacher] to explain it in clear details [to learners] particularly the benefits of VMMC… The main topic in youth clubs is HIV&AIDS so I think if HSAs and teachers were oriented we can pass on the knowledge when we are with the youth clubs*,” **[FGD, Service Providers – Dowa].**

#### What are the expected effects (or impact) of messages on VMMC services on the audience and policy?

Regarding the expected effects (or impact) of VMMC communication interventions and the VMMC program in general, the study established that the anticipated effects were at four different levels: at personal level, at household level, at community level and at policy level.

At personal level, interventions were expected to create awareness of VMMC and how it contributes to HIV prevention in heterosexual men, cervical cancer (among women) and other sexually transmitted infections. There was also an expectation of increased preventive post-circumcision behaviour among VMMC clients:

*“…one man did not observe the six weeks -sexual-abstinence window like he was instructed by the service provider. They resumed sex earlier and he developed complications,”***[KII, Service Providers, Chowe – Mangochi].**

In Dowa, the overall anticipated impact of VMMC communication interventions was the reduction of cervical cancer cases that were considered to be on the rise. One sex workers’ expectation had this to say:

*“The main issue is reduction of diseases; it is the same when the hospital is giving counseling on condoms, they say ‘every man should abstain from sex. However, if they fail then using a condom is Plan B’. It is the same with circumcision. It is Plan B,”***[FGD, Commercial Sex Worker - Dowa].**

In Dowa, there was also fear that VMMC communication interventions could also lead to rising culture of promiscuity that could lead to increased HIV prevalence: *“It [VMMC] is encouraging promiscuity among the youth here because if one boy is told that he is very sweet by a girl he wants to sleep with every girl…,”***[FGD, Service Provider – Dowa].**

Community members and health personnel working in local health facilities wanted to see household leaders, particularly mothers and clan leaders, to mobilize adult men and boys under their influence for services since they were primary producers of health. Sexual partners, mothers, aunties and grandmothers were considered to have the duty of motivating male members of their households to access services. They were also expected to support circumcised males to adopt post-circumcision prevention measures:

*“…females are the ones who go to the hospital… when the mother and other females are taught and understand [advantages of VMMC] it will be easy for the child to get circumcised,”***[FGD, Community Leaders, Dowa].**

Some traditional circumcisers were still reported using one razor blade or knife to circumcise more than one initiate. But, change had already started happening at community level since VMMC communication intervention rolled out in Mangochi:

*“Two weeks ago I was on a tour … Angaliba themselves were asking us: ‘Please, government should send us circumcision knives for use in initiation camps*,” **[IDI, Senior Chief – Mangochi].**

In Mangochi, informants felt that interventions enabled Yao Muslims practice jando as prescribed in the Holy Quran. One Yao Islamic cleric posited that VMMC already contributed to the demystification of male circumcision which, in the Yao culture, had been shrouded in secrecy from women, children and uncircumcised males for centuries: *“…I am very pleased because what is happening now [in clinics] is real jando as prescribed by the Islamic faith.… VMMC fully agrees with Islam,”***[KII, Religious Leaders - Mangochi]**.

In Dowa district, gatekeepers also expected non-formal institutions such as the *gulewankulu*^9^ supporting the VMMC campaign through information dissemination. They also expected it to lead in the reduction of cancer-related illness and deaths and to harness VMMC clients as advocates for services in their respective communities. In Dowa interventions were also expected to lobby for a more stable availability of funds to support community outreach programs: *“In the past we used to go on outreach programs and we would discuss (VMMC) with community members… We have no money for outreach programs,”***[FGD, Service Providers– Mponela, Dowa].**

Although they completely rejected the proposals to adopt male circumcision as a standard cultural practice in *gulewankulu* bases called *dambwe*^10^, the greatest change was that the Chewas were, nonetheless, ready to use *gule wankulu* characters as crowd puller to community meetings to ensure that VMMC messages quickly diffused into communities. *“…They can spread the message via songs since gule wankulu characters have a special talent when it comes to composing songs,”***[FGD, Community Leaders – Dowa].**

Informants both in Mangochi and Dowa also wanted to see secondary facility (i.e. district-level-hospital) circumcisers and community-level-hospital (i.e. local health facilities) personnel working together when carrying out circumcisions. To achieve this, informants were of the view that the district health system needed to ensure that there was adequate coordination and collaboration by all key stakeholders in the VMMC service delivery value chain in each district. *“If, on average, they had at least enrolled one nurse or clinician from each rural facility in the district …These could have been assigned to promote VMMC services at community level,”***[KII, Service Provider, Mangochi].**

Another service provider in Dowa : *“The job is done by the top dogs so… we can’t follow-up because we don’t know how they counsel [clients]. Maybe there isn’t any follow-up [in their plans],”***[FGD, Service Providers -Dowa].**

Informants also complained that VMMC circumcisions that were offered at health facilities lacked privacy for clients. They expected this to change if the program would be successful. In Mangochi, due to lack of a proper transport arrangements, older men were sometimes being ferried from rural areas to the secondary facility on the same open lorry with boys as young as 5 years. At one facility in Dowa, informants reported that circumcisions were taking place in a room whose entry was inside the maternity ward such that adult men shunned services. Many parents who were willing to get their sons circumcised shunned services.

“*… they had no choice due to lack of proper space … [VMMC clients] had to go past a group of pregnant women [in the maternity wing]. Boys were shy. The room should have [had] two doors so that after getting circumcised they should use the other door…*,” **[FGD, Service Providers – Dowa].**

At two facilities in Mangochi, informants told these researchers that VMMC had not been integrated into other services offered by facility and no special day or time had been allocated to these services since services were just being provided when circumcisers came all the way from Mangochi District Hospital. The other challenge both in Mangochi and Dowa was the long distances to service points. In the FGD with VMMC clients in Dowa said: *“Hospitals are very far from our area so it costs a lot of money to go there and sometimes we don’t have the money,”***[FGD, VMMC Clients – Dowa]**.

## Discussion

Both the Chewa and Yao people performed rites of passage with the same purpose of shaping the character of boys and transitioning them from childhood to adulthood. Nonetheless, s they differed in the rituals they performed for both boys’ initiation rites. While the Chewa initiated boys throughgulewamkulu the Yao people initiated them through *jando* where male circumcision was administered both for cultural and religious beliefs. The negative attitude to VMMC observable among the two ethnic groups is the major reason why there is low uptake of VMMC services both among the Chewa people of Dowa and the Yao people of Mangochi. They feared that the procedure was in conflict with their cultural identities, traditions and value systems. In particular, both data collection communities perceived VMMC as lacking in character formation which was the main drive behind rites of passage rituals in their areas.

However, the two cultures also differed on motivators for VMMC services. A fraction of the Yao people accepted VMMC because they did not have to pay anything to access services, except meeting transport costs, reduced pain, quicker healing of wounds, reduced risk of contracting HIV due to the use of sterilized razor blades and receiving services in a hygienic environment. Some Chewa people, on the other hand, were motivated to access VMMC services mainly because of its perceived ability to protect women from cervical cancer.

On culturally-preferred communication approaches, generally, both study communities opted for interpersonal face-to-face communication approaches since they gave them the opportunity to interface with health promoters and provide real-time feedback on the messages that they disseminated. The study also established that since the two ethnic groups were different from each other culturally and religiously such that, although they had similar communication needs in many respects, these two groups had different communication needs. This phenomenon suggested that communication programs should not be generic but must be designed to address the specific needs of each treatment group.

Regarding the **source of VMMC messages**, informants in both study groups posited that trained health workers were preferred. Health workers were preferred to disseminate such information because they were considered trained and well experience in disseminating health information hence expected to possess the required competence to carry out the task of reaching out to various audiences with VMMC messages. This finding is also consistent with other studies that have been conducted in Malawi and elsewhere on VMMC communication and have shown that health personnel are among the most trusted and preferred sources of information among males [12, 31]. The Ministry of Health in Kenya, for example, prioritized capacity building of health personnel including those involved in health communication [32]. It was intriguing, however, to learn that much as informants preferred health workers as sources of VMMC messages, study participants both in Mangochi and Dowa were of the view that community entry must be done in consultation with leaders of their communities. Thus, iformation delivery must take place in the presence of chiefs or anyone delegated to stand in.

The study demonstrated that health workers needed to work with chiefs in order to create a platform on which the message can be disseminated to the maximum number of people in the area. The contact between the health experts and traditional leaders provides the first opportunity for establishing a shared vision between health experts and the target audience. This suggest that the starting point for designing a good health communication campaign is the establishment of a shared vision between the health promoters and the target audience [33]. This finding agrees with Lozare et al. who positulated that since the primary producers of health are the people themselves there is need to engage them in the most meaningful way [33].

In the Malawi setting, when the chief and the rest of the cultural structures reject a message, the whole community follow suit. Generally, informants approved the current practice where health workers were at the center of message dissemination. However, they expressed worry that, in the current set up VMMC workers from the district hospital or the NGO partners, invaded their villages and communities with loud hailing before adequately engaging traditional leaders, cultural gatekeepers and without building partnerships with cultural structures that carried out similar work in the local community. They further deplored the failure by health experts to hold community meetings through which community members would have a chance to ask questions and seek clarification on aspects of VMMC that were not clear.

Regarding **messaging**, informants wanted messages that were contextual and unambiguous and promoted HIV prevention. They expressed worry that although VMMC had the potential of positively contributing to the reduction of HIV prevalence sexually explicit messages, which were frequently heard in the media, were perceived to have the potential of increasing promiscuity among the youth, a phenomenon that could potentially worsen the HIV situation in their areas. This notion reflects the general Malawian worldview that the function of sex surpasses the procreation and sensual function as it connotes survival of the extended family, clan and tribe [34, 35]. In his writings Augustine Musopole argues that, in Africa sexuality is one of those vital forces that make life secure, meaningful and worthwhile [35]. He, therefore, advises that it is largely a taboo to openly discuss sex and sexuality across many African cultures where adolescents were separated from the community and taken to the bush to be taught sexual rituals, moral values, and cultural norms [34, 36]. In this study informants generally agreed that messages that put emphasis on the sexual pleasure as a benefit of VMMC services were not welcome to the majority of Malawians. Both in Dowa and Mangochi, older informants expressed concern that sexual pleasure was dominating the VMMC messaging instead of focusing on HIV prevention outcomes. These findings add value to the conclusions of the study in Malawi by Patrick Makono et al. who observed that lack of a proper research prior to the design of Malawi’s *Ndife Otsogola* VMMC brand, resulted in messages that failed to effectively promote the uptake of VMMC services among men and boys in Malawi [37].

Both study sites preferred messages that focused on health benefits of VMMC such as the prevention of HIV and cervical cancer. The Chewa people, on one hand, were almost complacent about their vulnerability to HIV infection because they did not practice polygamy and their instruction, both in the cultural and religious initiation rituals, was perceived to be strong on its emphasis on sexual abstinence. They, therefore, felt that if VMMC messages were to appeal to the majority of them, they needed to create an awareness of how male circumcision contributed to the reduction of deaths due to cervical cancer among women. Yao informants, on the other hand, posited that both HIV and cervical cancer prevention was a motivating factor. They, therefore, preferred messages that created the awareness, that facility circumcision service reduced pain, ensured early healing of the wound and the risk of post-op complications. They also preferred messages that portrayed VMMC services as being cheaper and more hygienic than traditional circumcision. Findings on the need for benefit-centered messages and their indispensability in promoting the uptake of VMMC services are similar to outcomes of other studies conducted in Malawi. For example, Mhagama et al. and Makono, et al. established that men and boys will opt for medical circumcision after obtaining benefit-centered information from medical personnel and personalized information from their peers [12, 37]. In a study conducted in KwaZulu-Natal in South Africa by George Gavin et al. established that knowledge-rich personalized-information was a vital facilitator of service uptake in South Africa [38]. Another study carried out in Rakai, Uganda, by Ssekubugu, et al., demonstrated that males acted on VMMC messages that emphasize on health benefits [6].

Regarding **channels of communication**, radio came out prominently as a common channel that government agencies and NGOs were using to disseminate messages on VMMC services. However, as Tilson et al. have noted, one key obstacle to effective communication in the past pertained to conceptualizing communication as a simple one-way transmission from the source to a receiver with the intention of producing some effect [39]. Many study participants pointed out that radio and loud hailing was not interactive enough because it did not provide them with an opportunity to ask questions and seek clarification on the messages that it delivered to listeners. They, instead, preferred community-based interpersonal communication to any other form of communication. For example, the Chewa people of Mwancheka area, in Dowa district, preferred approaches that brought families and communities together. Messages could therefore be delivered during funeral receptions, religious functions, drama performances and gule wankulu functions, just to mention a few. One community leader said: *“Even when there is a funeral the chief can stand [up] and deliver information on VMMC.”* Study participants in Dowa were completely opposed to mainstreaming male circumcision into the male-dominated *gulewankulu.* However, they were open to the use of *gulewamkulu* to accelerate the dissemination of messages on VMMC services in their communities. It is for this reason that communication channels that enabled families and communities to collectively have direct access to VMMC messages in an interactive manner were preferred. This view point was consistent with Benjamin Lozare’s assertion that since the households and communities were the primary producers of health, communication approaches that sought to empower such units needed to be prioritized [33].

About the **audience**, the key problem with VMMC campaigns in Malawi, according to informants from both study ethnic groups, was the focus on the individual male rather than every ‘individual citizen’ of the community in the VMMC messaging. It was recommended by study communities that VMMC messages must target the man, the boy, the woman, and all social structures in the social-ecology. They argued that the message must be directed to close family members, peers of eligible men and boys, sexual partners and caregivers who happen to be females generally. Decisions either to access VMMC or not are not entirely personal for men or boys. Like it was discovered in Uganda, this study, both in Dowa and Mangochi, found that for married men, female sexual partners had a strong say on the decision a man makes [40]. This finding also agrees with studies conducted in Papua New Guinea, Zimbabwe and Botswana. In these studies, it was observed that women played a significant role to improve uptake of voluntary medical male circumcision (VMMC) and in the post-op behaviors of the client [41]. These studies established that women could motivate men, particularly their sexual partners, to access VMMC services. Women were willing to engage their husbands on the benefits of VMMC such as improved penile hygiene, reduced risk of HIV transmission and other sexually transmitted infections, as well as increased sexual satisfaction [41]. In the matrilineal marriage system which is generally practiced in Malawi and in the study areas i.e. Mangochi and Dowa, extended family members particularly uncles, aunts, and grandmothers, as well as clan heads *(eni mbumba)*, have a great deal of influence in decision making processes at household level [36, 42]. Among the Chewa people of Dowa for example, the woman generally moves to live in the man’s village under a practice called *chitengwa*^11^ while among the Yao of Mangochi, the man is expected to move and live in the woman’s village under a practice called *chikamwini*^12^. This means that apart from aiming messages at eligible men and boys, the campaign must also aim at reaching the entire community, particularly women, if access to services is to improve.

This study has unearthed mixed preferred **effects** of VMMC messages. These preferred *effects*, according to the Social-Ecological Model that was used in identifying informants to this study, manifested at four different levels of the social ecology namely: at individual level of a circumcised male; at household level; at community or society level and, finally, at policy level. The preferred effect of VMMC messages on the primary audience, adult men and boys, is sixfold. First, is an awareness and an understanding of VMMC with a view to demystify the concept of voluntary medical male circumcision - a phenomenon that would lead to increased uptake of services, reduced transmission of HIV, cervical cancer among women and girls and sexually transmitted infections. Secondly, an assurance both among Christians and Muslims that VMMC had not been introduced neither to contradict the Bible or the Qur’an respectively nor for political reasons since its purpose is purely biomedical. Thirdly, informants mentioned increased post-circumcision preventive behaviour among clients. Fourthly, they expected increased sexual pleasure and a reduced risk of complications due to early sexual resumption and a reduced fear of pain particularly among adults. At household level informants expected two key impacts. First, mothers and female household members, as primary producers of health [33], were expected to use their ‘authority of matrilineality’ to encourage men to access services and get their sons, particularly infants and pre-adolescents and adolescents, circumcised at the facility. Secondly, clan leaders were expected to play their rightful role as *mwini mtundu*^13^ or *mwini mbumba*^14^, by encouraging men, pre-adolescents and adolescents to get circumcised at the facility and supporting them adequately after accessing services. At community level in Mangochi, informants expected to see the reduction of three risky practices: adult sexual instruction that initiates as young as 5 or 6 years were exposed to in rituals called *kusasa fumbi*^15^ for girls and *kutaya mafuta*^16^ for boys; sexually explicit language and songs and the use one razor blade or knife to circumcise more than one initiate by traditional circumcisers. In Dowa, community leaders and gatekeepers expected to see the *gulewankulu*^17^ community support the cause of the VMMC campaign by disseminating messages in their own communities; the increased adoption of VMMC services by adult men who were said to be reticent on access to health services generally. Lastly, the key effect that informants expected from VMMC communication programming was community development as a result of good health at household level.

These authors have noted, however, that *feedback* is the key missing link in Laswell’s Transmission Theory since the theory discusses communication purely as a linear process on the assumption that all there is in a communicative process are sources of information, messages, the audience, medium or channel through which sources speak, and effects. This observation was also made by Peng Wenxiu [30]. This was not complete because, for real effect to occur, like Lozare puts it, the communicator (source of the message) and the audience (the consumer of the message) need to have a *common vision* or *understanding* for true behaviour change to occur [43]. For this to happen there is need for those involved in VMMC communication to appreciate that communication is not a one-way lane but a two-way road with interaction between the communicator and the audience. It is for this reason that this study concluded that *community engagement* and *interpersonal communication* which provide room for real-time *feedback* in any communicative event are the preferred communication interventions for VMMC services among Yaos and Chewas of Southern and Central Malawi respectively. Participants both in Mangochi and Dowa raised a serious concern that health practitioners involved in the promotion of VMMC services in their districts did not adequately engage them in their villages. This, they observed, robbed them of the chance to ask questions and to seek clarification on matters that concerned them or were not clear regarding facility circumcision.

### Study strength

1. The study has clarified five key elements in the communication continuum of VMMC interventions including source, audience, message, channel, effect and feedback and has demonstrated the place of communication in health promotion.
2. The study has demonstrated the indispensability of interpersonal communication health communication with a special emphasis on the need for real-time feedback.
3. It has also provided a platform for further inquiry into health promotion programming particularly in the use of Laswell’s Transmission Theory in health communication in the African context.

### Limitations

1. Being a by-product of a purely qualitative study, this report lacks statistical basis upon which conclusions could effectively be grounded.
2. Although the principal investigator sought the services of two Yao insiders (a male and a female) and one Chewa insider (a female in-training health worker) to collect and support data analysis, he is aware that his own personal biases may have been introduced in the data analysis and presentation of findings. To counter the bias the data analysis approach was guided by the thematic framework approach whereby rigorous reading and re-reading of each transcript was undertaken with the view to getting familiar with the content of the transcripts. The repeated review of transcripts informed the identification of codes which were then classified into sub-themes which were further synthesized into bigger themes. This effectively minimized the possibility of introducing biases into the content. The codes were developed from the emerging issues from the responses as was required by the study questions which fully underwent a review and approach of the National Committee on Research Ethics in the Social Sciences and Humanities (NCRSH). In this regard, the approach to data collection and analysis was guided by the stipulated ethical obligations by a competent review IRB board implying that the researchers were guided and made sure to adhere to the stipulated ethical obligations and principles of thematic framework approach which is the analysis methodology followed. Further to this, this is a product of team work involving the corresponding author and a team of co-authors who participated in the drafting of manuscript and review of the same suffice to say that co-authors were from different cultural backgrounds.

## Conclusion

The key finding of the study is that community stakeholder engagement, complemented by various interpersonal communication and small-group approaches such as village town-hall meetings, drama, religious and cultural gatherings (including gulewankulu festivities for the Chewa people), is the preferred communication approach both in Yao and Chewa communities. The main reason is that apart from its strong emphasis on exchanging knowledge between the source and the audience in a human-to-human fashion, community stakeholder engagement and interpersonal communication allow the communicator and the audience to foster a *common vision* which, according to Ben Lozare, is a prerequisite for behavioral change. This study also established that one critical element that must be created in every health communication situation is *feedback*. It is this element that makes the development of a *common vision* between the health communicator and the target audience. It is in this context that true adoption of a desired behavior can be achieved.

## Data Availability

The datasets generated and analyzed during the current study are not publicly available due to confidentiality. However, they are available from the corresponding author on reasonable request.

## Footnotes

1 A person considered to be well grounded by experience and training in traditions, values and practices of the Yao people.

2 This is the Yao initiation camp where boys are circumcision. This word is interchangeably used with *jando*.

3 *Syphilis*,

4 *Chancroid*

5 *Gonorrhea*

6 *A traditional Yao initiation camp*

7 *A catechist or lay pastor in the Catholic Church*

8 *An initiation camp for Yao boys also called ndagala.*

9 *Translated as ‘great dance’, gule wankulu is, as defined by Claude Boucher, a “dance ritual of extraordinary complexity and richness” that is common among the Chewa people of Malawi, Mozambique and Zambia. It is also called Nyau. A cultural grouping in the Chewa society that is responsible for rites of passage for both boys and girls. It is also associated with masked characters and strict discipline.*

10 *A cultural base shrouded in secrecy, usually located in the gravesite or thick forest where Chewa men and sometimes women create various gule wankulu characters and rehearse for public performances.*

11 *The practice prevalent among the Chewa people of Dowa, whereby the married woman sometimes leaves her home village to live in the husbands home village.*

12 *The practice common among both the Yao and Chewa people of Mangochi and Dowa respectively, whereby the married man leaves his home village to live in the wife’s home village.*

13 *This is the senior most maternal uncle who is the final decision maker in a Yao household.*

14 *This is the senior most maternal uncle who is the final decision maker in a Chewa household.*

15 *This is a ritual where a Yao girl is expected to have her initial sex as a way of removing her virginity after undergoing initiation. These researchers also heard about this practice during interviews with Chewas of Dowa.*

16 *This is a ritual where a boy is expected to have his initial sex as a way of removing excess fat from his body after undergoing initiation. Although the practice is common in Yao societies, Chewas too mentioned this practice is passing.*

17 *Translated as ‘great dance’, gule wankulu is, as defined by Claude Boucher, a “dance ritual of extraordinary complexity and richness” that is common among the Chewa people of Malawi, Mozambique and Zambia. It is also called Nyau. A cultural grouping in the Chewa society that is responsible for rites of passage for both boys and girls. It is also associated with masked characters and strict discipline.*
